# B cell-rich non-neoplastic sentinel lesion preceding primary central nervous system lymphoma

**DOI:** 10.1186/s13000-018-0717-9

**Published:** 2018-06-05

**Authors:** Rodrigo Javier, Nawal Shaikh, Maciej S. Lesniak, Adam Sonabend, Roger Stupp, Amir Behdad, Craig Horbinski

**Affiliations:** 1 Departments of Neurological Surgery, Feinberg School of Medicine, Northwestern University, Tarry 2-705, 300 East Superior Street, Chicago, IL 60611 USA; 2 Departments of Neurology, Feinberg School of Medicine, Northwestern University, Chicago, IL 60611 USA; 3 Departments of Pathology, Feinberg School of Medicine, Northwestern University, Chicago, IL 60611 USA

**Keywords:** Primary CNS lymphoma, Diffuse large B cell lymphoma, Sentinel lesion

## Abstract

Primary central nervous system lymphoma (PCNSL) is an uncommon tumor in the brain. Although most PCNSL are readily diagnosed as diffuse large B cell lymphoma (DLBCL) on the first biopsy, very rare cases have been described in which the first detected intracerebral lesions are non-neoplastic, and are composed mostly of perivascular T cells, not B cells. This phenomenon is known as “sentinel lesions.”

## Case presentation

We describe a case of a patient who presented with dysarthria and blurry vision, and was found to have abnormal T2 hyperintensity and contrast enhancement along both internal capsules and basal ganglia. Biopsy of the right basal ganglia showed an extensive intracerebral perivascular infiltrate with abundant small, mature-appearing B cells that showed no evidence of clonality. After four months of monitoring, a new expansile lesion developed in the left middle cerebellar peduncle; biopsy of the new lesion showed unequivocal DLBCL, including clonal immunoglobulin heavy chain rearrangement.

## Conclusions

To the best of our knowledge, this is the first indication that pre-PCNSL sentinel lesions, previously reported to only show T cell predominance, can also contain large amounts of non-neoplastic B cells. This underscores the importance of considering PCNSL as the underlying disease when abnormally high levels of B cells are found within a brain biopsy, even when there is no flow cytometric, immunohistochemical, or molecular evidence of clonality.

## Background

Primary central nervous system lymphoma (PCNSL) is very rare, with an age-adjusted incidence rate of only 0.44/100,000, less than 2% of all non-Hodgkin lymphomas throughout the body [1]. The vast majority of PCNSL is diffuse large B cell lymphoma (DLBCL) that is CD20/MUM1 positive. CD10 may be present in some cases, and many tumors show Bcl6 expression, even if only focally [2]. Unlike most systemic DLBCL, PCNSL often expresses high levels of Myc, yet typically lacks *MYC* rearrangement [3]. Most PCNSL cases are sporadic, and are not linked to anything other than advanced age and male gender [2, 4]. Radiologically, PCNSL tends to be rather symmetrical, with involvement of the bilateral white matter and corpus callosum by T2 iso-to-hyperintense, contrast-enhancing lesions that fluctuate over time [2]. Epstein-Barr virus infection is associated with DLBCL in immunocompromised patients, is not considered to be part of PCNSL pathology, and has decreased in frequency in the era of effective anti-HIV therapies [2].

Whereas most PCNSL present as DLBCL histologically, rare examples of non-neoplastic perivascular T cell-rich sentinel lesions have been described that precede the onset of frank PCNSL. Herein we describe a unique PCNSL case where the sentinel lesion within the cerebrum contained large amounts of non-neoplastic B cells.

## Case presentation

**Fig. 1:**
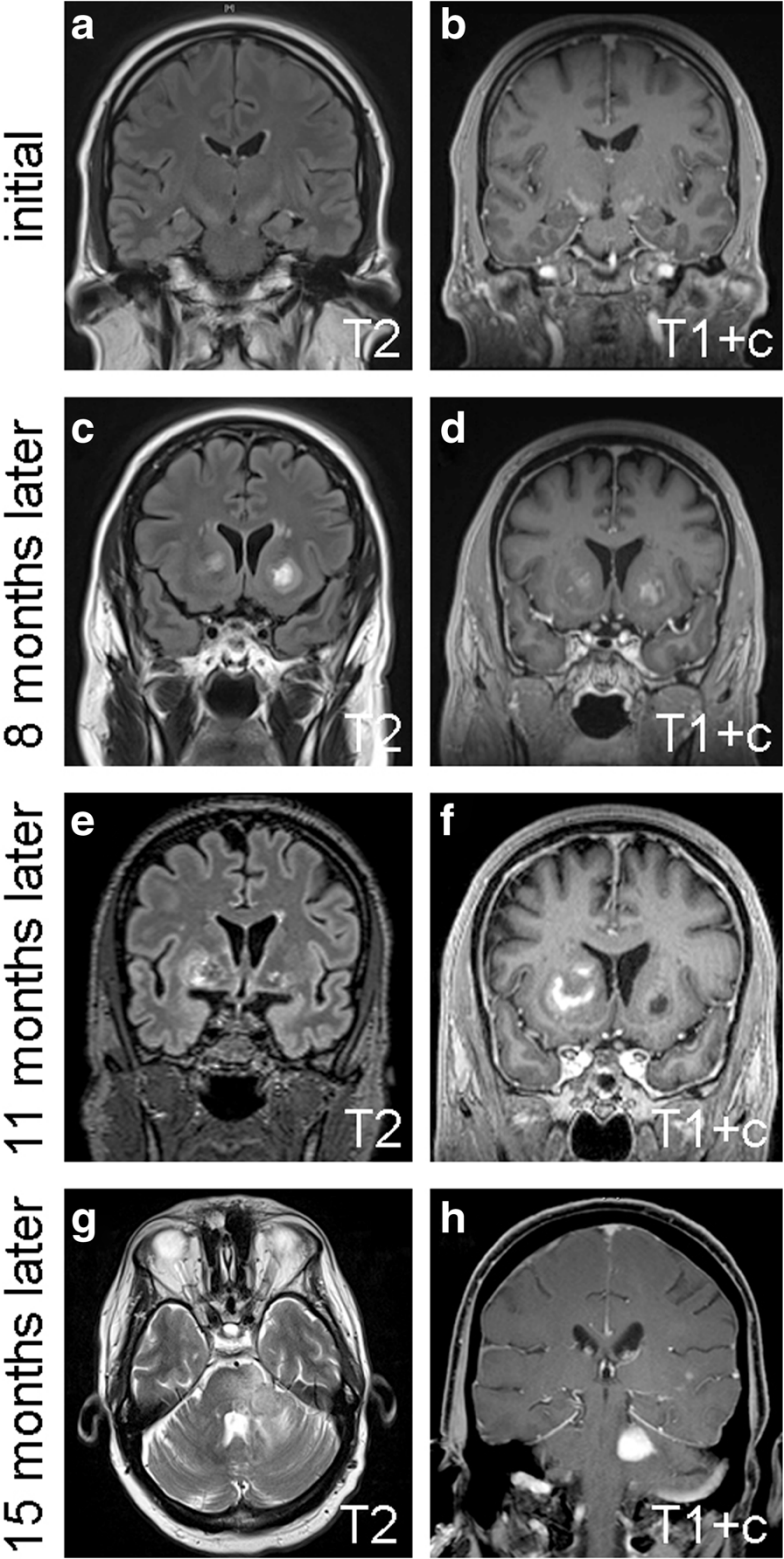
Serial MRIs. The initial set of MRIs showed T2 hyperintensity and some contrast enhancement in the bilateral internal capsules (**a**, **b**). Eight months later, a second set of MRIs showed new enhancing lesions in the bilateral basal ganglia (**c**, **d**). Eleven months after initial presentation, the right basal ganglia lesion appeared to grow, whereas the lesion in the left basal ganglia diminished (**e**, **f**). In the final set of MRI, 15 months after initial presentation, the right basal ganglia lesion was unchanged (not shown), and a new enhancing mass appeared in the left middle cerebellar peduncle (**h**). c = contrast

The patient was a 60 year-old previously healthy man who presented to an outside institution with a rapid onset of dysarthria without aphasia, as well as blurred vision in his right eye without ptosis. The patient had a history of high blood pressure (controlled with losartan) and acid reflux, but no recurring infections, headaches, abnormal clotting or bleeding, night sweats, dysphagia, or weakness. At the outside institution, a full battery of laboratory testing, including complete blood count, complete metabolic profile (CMP), erythrocyte sedimentation rate, vasculitis panel, autoimmune panel, and myasthenia gravis panel, was negative. A stroke was therefore initially suspected, but magnetic resonance imaging (MRI) showed T2 hyperintensity tracking along both corticospinal tracts from the corona radiata through the posterior limb of both internal capsules and cerebral peduncles, as well as some matching contrast enhancement (Fig. 1a-b). Although nonspecific, this was originally felt to be most consistent with a neurodegenerative disease such as amyotrophic lateral sclerosis (ALS) or other metabolic disorder. At a second external institution, electromyography was obtained that ruled out ALS.

The patient remained fairly stable for the next several months, and declined follow-up appointments and imaging. Four months after initial presentation, he developed bilateral blurry vision and leg weakness, yet still declined follow-up care until arriving at our institution 8 months after his initial presentation. An MRI at that time showed new enhancing lesions in the bilateral basal ganglia (Fig. 1c-d), raising concern for CNS vasculitis. Yet aside from a mild elevation of antinuclear antibodies, his serum protein electrophoresis, vitamin B12, vitamin D, folate, and repeat autoimmune and CMP panels were all within normal limits. Computerized tomography of the patient’s chest, abdomen, and pelvis were all negative for lymphadenopathy. Flow cytometry on a cerebrospinal fluid sample was negative for atypical lymphocytes; only 1% of the lymphocytes present in the cerebrospinal fluid were CD19+ B cells.

**Fig. 2:**
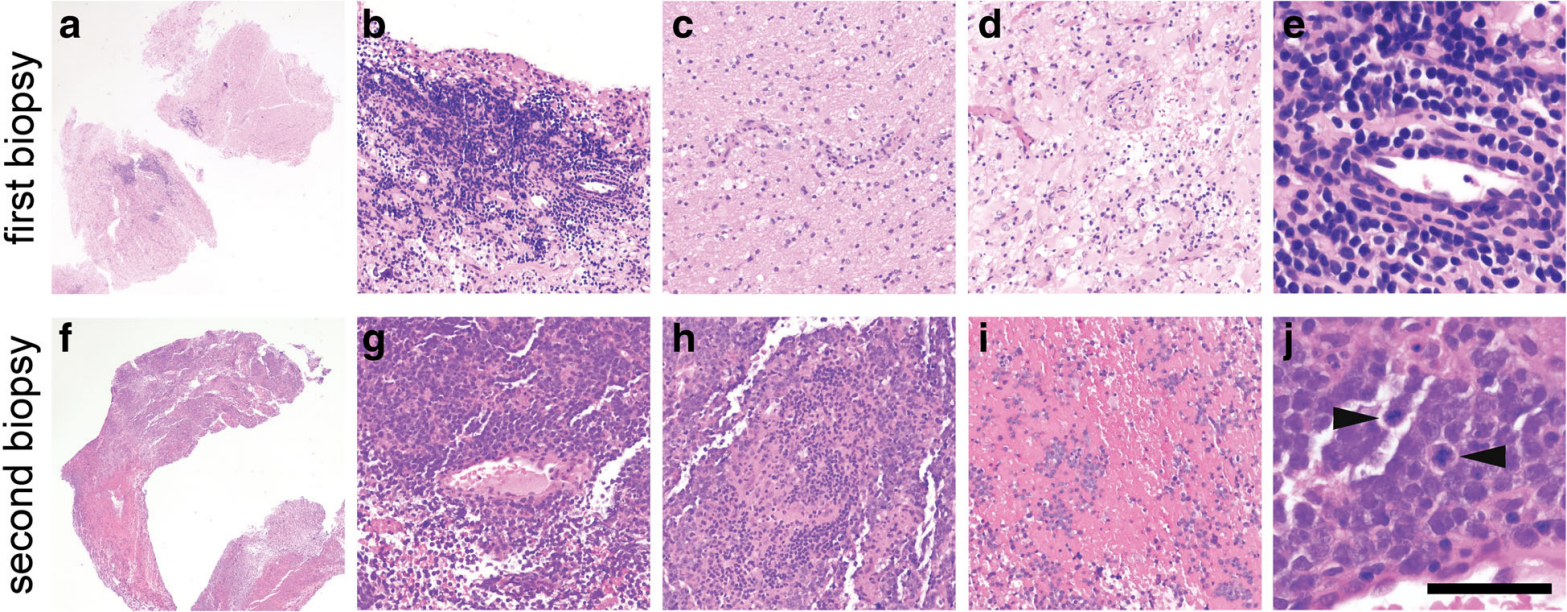
Hematoxylin and eosin (**H** & **E**) features of both biopsies. In the first biopsy of the right basal ganglia lesion (**a**,**e**), abundant mature-appearing lymphocytes were present in a mostly perivascular distribution. In the second biopsy of the left middle cerebellar peduncle lesion (**f**-**j**), there were sheets of neoplastic cells with large nuclei and numerous mitoses (j, arrowheads). Scale bar = 2 mm in a & f, 200 μm in b-d and g-i, and 50 μm in e & j

**Fig. 3:**
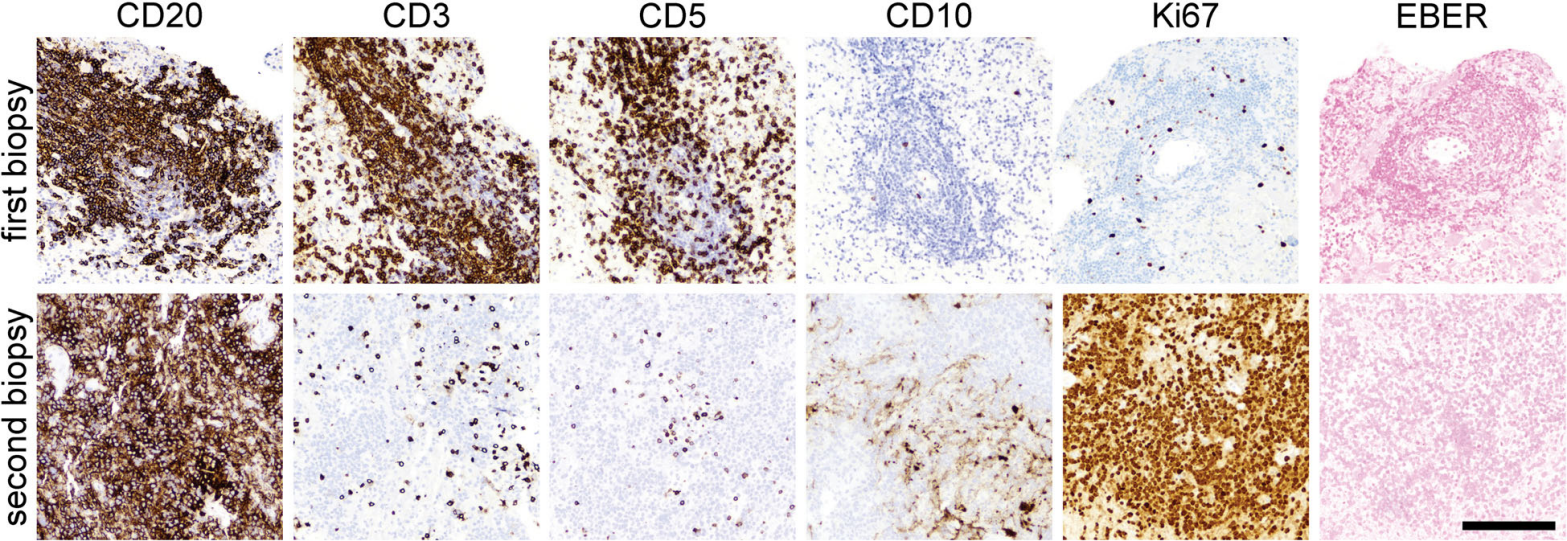
Comparative immunohistochemistry between the first and second biopsies. See “Case presentation” section for description. Of note, the vessel shown in the CD20/3/5/10 panels from the first biopsy was adjacent to the vessel shown in the Ki67/EBER panels, due to the original vessel diminishing on deeper sectioning. Scale bar = 200 μm

Up to this point, the comprehensive workup had been inconclusive, so no treatment had yet been administered, including steroids. But when another MRI showed further progression of the right basal ganglia lesion 3 months later (11 months after initial presentation, Fig. 1e-f), a stereotactic biopsy of the right basal ganglia was taken in an effort to establish a diagnosis. The tissue showed brisk perivascular and parenchymal lymphocytic infiltration (Fig. 2), but no evidence of vessel wall damage. All the lymphocytes appeared small and mature, with no mitoses anywhere in the biopsy. While immunohistochemical studies showed a strikingly high number of CD20-positive B cells, relative to what is typically seen in inflammatory diseases of the brain, as well as large numbers of admixed CD3/5-positive T cells, the Ki67 proliferation index was only 5% (Fig. 3). Other stains, including CD10 and EBV-encoded RNA (EBER) in-situ hybridization were negative (Fig. 3), as was Bcl1 (not shown). Luxol fast blue showed no lipid-laden macrophages or other evidence of demyelination (not shown). Flow cytometry performed on an unfixed portion of the biopsy was negative for abnormal clonal subpopulations, and molecular analysis was negative for immunoglobulin heavy chain gene rearrangement. The biopsy was then sent for external review by additional neuropathology and hematopathology experts, who concurred that a neoplastic process could not be proven.

Despite the lack of any definitive evidence of a B cell neoplasm on the initial biopsy, the high number of B cells still raised concern for a lymphoproliferative process. A working diagnosis of low grade B cell lymphoma was therefore discussed, with a differential including demyelinating disease with B cell infiltrates. Thereafter, the patient was prescribed 4 doses of rituximab over 4 weeks, along with dexamethasone, with no clinical improvement. Patient adherence to the medication was uncertain, and subsequent appointments and imaging were delayed.

**Fig. 4:**
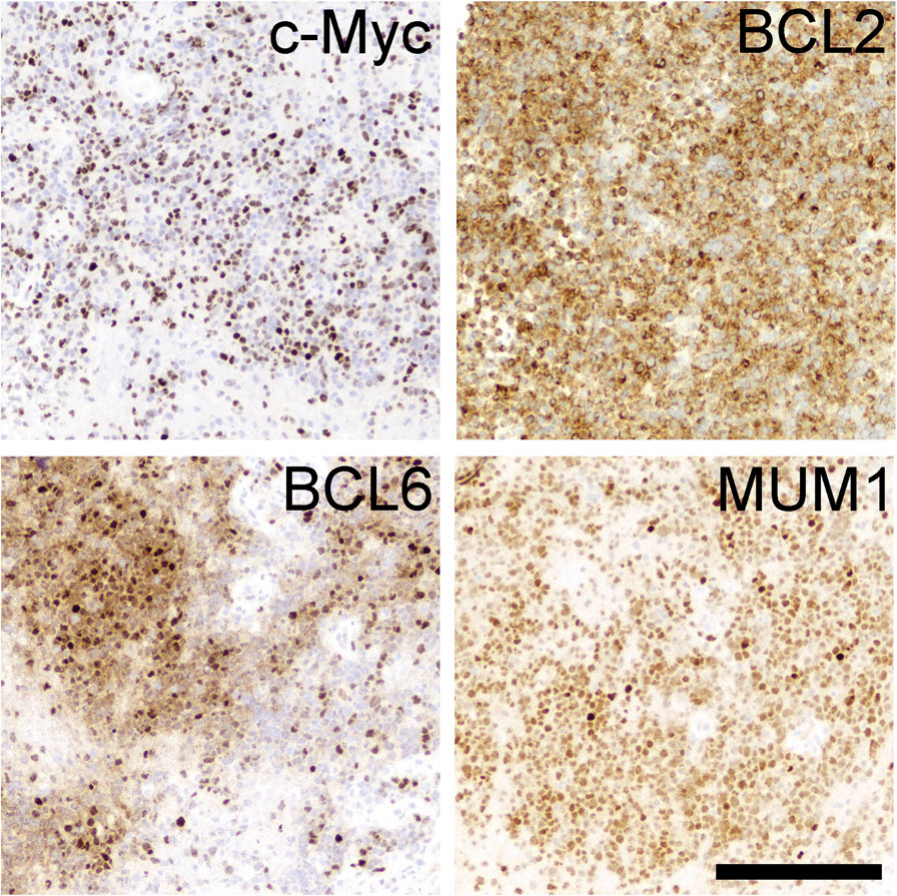
Additional immunohistochemistry from the second biopsy. See “Case presentation” section for description. Of note, most of the neoplastic cells in the biopsy were Bcl6 negative; the image shows the most positive region. Scale bar = 200 μm

Four months after the first biopsy (15 months post initial presentation), the patient developed unsteady walk, cheek and tongue numbness, post-meal emesis, slurred speech, and worsening bilateral leg weakness. A fourth MRI showed a new enhancing region in the left middle cerebellar peduncle (Fig. 1g-h), prompting a biopsy of the peduncular lesion. This time, the tissue showed an obvious malignancy, composed of cells with large, highly atypical nuclei and clumped chromatin, scant cytoplasm, and abundant mitoses (Fig. 2). Unlike the prior biopsy, the repeat biopsy showed a clear preponderance of CD20-positive B cells, as well as an extremely high Ki67 proliferation index approaching 100% (Fig. 3). MUM1 and cMYC were positive in 80 and 40% of the tumor cells, respectively, while Bcl2 was present in 80% of cells and Bcl6 was expressed by only 10–15% of the cells overall (Fig. 4). CD5, CD10, and EBER were all negative in the neoplasm (Fig. 3). Immunoglobulin heavy chain gene PCR showed clonal rearrangement, whereas a fluorescent in situ hybridization test for *MYC* rearrangement was negative (not shown). No flow cytometry was done on the second biopsy. Subsequent computerized tomography showed no evidence of systemic lymphadenopathy.

Based on the new diagnosis of a primary CNS diffuse large B cell lymphoma, the patient was administered high-dose methotrexate, temozolomide, and rituximab. This regimen failed, and the patient continued to progress such that, 19 months after his initial symptoms, he was suffering from dysphagia, aspiration pneumonia, and hypoxia.

## Discussion and conclusions

The term “sentinel lesion” was first coined in 1996 to describe extremely rare cases in which a non-neoplastic intracerebral perivascular inflammation occurs before the onset of clinically overt PCNSL [5]. Prior to the current case, 12 examples of sentinel lesions had been described in the literature, revealing an emergence of distinctive clinical, histologic, and radiologic patterns (Table 1). Like PCNSL in general, patients with PCNSL-associated sentinel lesions have a median age of 57. Although the male:female ratio for all PCNSL is 3:2 [2], sentinel lesions appear to be more likely in females. None of the sentinel lesions have occurred in patients with prior immunocompromised conditions, and none of the cases in which EBV was tested have been positive. All sentinel lesions have shown T2 hyperintensity and various degrees of contrast enhancement that usually fluctuated over time prior to the onset of frank PCNSL. In reports including detailed histology, all found a perivascular pattern of lymphocyte-rich inflammation, often accompanied by demyelination. In all such cases, inflammation was mostly composed of T cells, with only rare mature B cells and variable macrophages. The median time between sentinel lesion biopsy and a diagnosis of PCNSL was 9 months, although three patients had extended intervals of 2–4 years before PCNSL. With one exception [6], none of the PCNSL biopsies were taken from the same site as the sentinel lesions. To the best of our knowledge, the current case is the first example of a PCNSL-associated sentinel lesion containing large numbers of mature B cells, yet with no evidence of clonality.

**Table 1:** Reported PCNSL cases preceded by sentinel lesions

Reference	Age	Sex	Immune status	Steroids prior to first biopsy?	Demyelinating?	Predominant lymphocyte	Time between sentinel lesion biopsy and PCNSL diagnosis
Alderson et al. 1996 [5]	58	F	Competent	Yes	Yes	T cell	7 months
	49	F	Competent	No	No	None (no inflammation)	11 months
	54	F	Competent	Yes	No	T cell	9 months
	57	F	Competent	Yes	Yes	T cell	9 months
Kuhlmann et al. 2001 [21]	65	M	Competent	Yes	Yes	T cell	9 months
Ng et al. 2007 [16]	29	F	Competent	Yes	Yes	not available	48 months
Husseini et al. 2012 [18]	59	F	Competent	No	Yes	T cell	30 months
Yamamoto et al. 2014 [22]	70	M	Competent	No	Yes	not available	3 months
Taieb et al. 2014 [13]	58	M	Competent	No	No	T cell	24 months
Lu et al. 2016 [20]	44	F	Competent	No	Yes	T cell	5 months
Kvarta et al. 2016 [6]	57	F	Competent	Yes	Yes	T cell	6 months
Current case	60	M	Competent	No	No	B cells > T cells	4 months

In general, inflammatory processes within the brain are composed predominately of T cells, and at most contain only a few scattered B cells. Even very florid cases of intracerebral inflammation, as seen in tumefactive multiple sclerosis or acute disseminated encephalomyelitis, contain far more T cells than B cells, although the latter do contribute to autoimmune diseases within the brain [7, 8]. Therefore, when abnormally large numbers of B cells are found in a brain biopsy, exceeding T cells, a lymphoid neoplasm is the most likely cause. Yet in the current case, the first biopsy only showed mature-appearing B cells, raising the possibility of a low-grade B cell neoplasm. Intracranial low-grade B cell lymphoproliferative disorders and lymphomas are very rare, and when they do occur, they are far more common in the meninges than in the cerebral parenchyma [2] and show some sign of clonality, e.g. light chain restriction on flow cytometry or heavy chain gene rearrangement via PCR [9–12]. The first biopsy from this patient produced no such evidence. Lymphomatoid granulomatosis (LG) is an angiocentric lymphoproliferative disorder that contains varying amounts of B cells, can progress to DLBCL, and rarely can originate in the brain rather than the more common sites, skin and lungs [13, 14]. Yet LG is defined by the presence of EBV and overt vasculitis with necrosis [15], whereas both biopsies from the current patient were EBER-negative and showed no sign of vessel wall damage or necrosis.

The pathophysiology of pre-PCNSL sentinel lesions remains unclear, though several hypotheses have been advanced. First, the non-neoplastic inflammatory cells may represent an immune response against clinically occult PCNSL, which only manifests when a subclone develops that can better evade the immune system [5]. For example, a young woman with presumptive demyelinating disease only developed overt PCNSL after giving birth, suggesting that pregnancy-related changes in her immune system allowed the PCNSL to finally emerge [16]. Second, the sentinel inflammatory lesions could represent paraneoplastic autoimmune disease caused by PCNSL, as has been described in systemic non-Hodgkin lymphomas and carcinomas from such sites as ovary, lung, breast, and thymus [6, 17]. To date, no data exist on the presence of well-known paraneoplastic antibodies like anti-Hu, -Yo, or -Ri in any patient with PCNSL and sentinel lesions. A third hypothesis is that, in rare instances of an intracerebral autoimmune disease, a pre-neoplastic B cell subclone may develop and eventually transform into full-blown PCNSL [6, 18]. Some lymphomas may develop from chronic autoimmune diseases in this manner, though the evidence for this is limited to systemic lymphomas, not PCNSL [19]. However, in all but one previously reported example of sentinel lesions and PCNSL, the overt malignancy appeared to develop at a completely different location within the brain from the sentinel lesions, as was the case in our current patient; one postmortem study even showed no evidence of PCNSL at the site from which the sentinel lesion was identified [6, 20]. Finally, some patients may have had PCNSL right from the beginning of their symptoms, but pre-biopsy corticosteroids caused selective apoptosis in PCNSL cells, leaving behind only the non-neoplastic immune components [2, 6]. However, as the current case demonstrates, there is no link between sentinel lesions and pretreatment with steroids (Table 1).

In summary, although it is not yet clear what causes rare PCNSL patients to develop non-neoplastic inflammatory sentinel lesions, this case expands the histologic range of such lesions to include large numbers of non-neoplastic B cells. PCNSL must therefore be retained in the differential diagnosis of older patients with T2 hyperintense, contrast-enhancing lesions, even when such lesions show a relapsing-remitting course and the initial biopsies show no sign of malignancy.

## Abbreviations

CMP: Complete metabolic profile
DLBCL: Diffuse large B cell lymphoma
EBER: EBV-encoded RNA
LG: Lymphomatoid granulomatosis
MRI: Magnetic resonance imaging
PCNSL: Primary central nervous system lymphoma
